# Fatigue factors and fatigue indices in SSVEP-based brain-computer interfaces: a systematic review and meta-analysis

**DOI:** 10.3389/fnhum.2023.1248474

**Published:** 2023-11-16

**Authors:** Maedeh Azadi Moghadam, Ali Maleki

**Affiliations:** ^1^Department of Biotechnology, Faculty of New Sciences and Technologies, Semnan University, Semnan, Iran; ^2^Department of Biomedical Engineering, Semnan University, Semnan, Iran

**Keywords:** brain-computer interface (BCI), steady-state visual evoked potential (SSVEP), fatigue, visual stimulation paradigm, quantitative indices

## Abstract

**Background:**

Fatigue is a serious challenge when applying a steady-state visual evoked potential (SSVEP)-based brain-computer interfaces (BCIs) in the real world. Many researchers have used quantitative indices to study the effect of visual stimuli on fatigue. According to a wide range of studies in fatigue analysis, there are contradictions and inconsistencies in the behavior of fatigue indicators.

**New method:**

In this study, for the first time, a systematic review and meta-analysis were performed on fatigue indices and fatigue caused by stimulation paradigm. We queried three scientific search engines for studies published between 2000 and 2022. The inclusion criteria were papers investigating mental and visual fatigue from performing a visual task using electroencephalogram (EEG) signals.

**Results:**

Attractiveness and variation are the most effective ways to reduce BCI fatigue. Therefore, zoom motion, Newton’s ring motion, and cue patterns reduce fatigue. While the color of the cue could effectively reduce fatigue, its shape and background had no effect on fatigue. Additionally, the questionnaire and quantitative indicators such as frequency indices, signal-to-noise ratio (SNR), SSVEP amplitude, and multiscale entropy were utilized to assess fatigue. Meta-analysis indicated that when a person is fatigued, the spectrum amplitude of alpha, theta, and $\left(\alpha +\theta \right)/\beta$ increase significantly, while SNR and SSVEP amplitude decrease significantly.

**Conclusion:**

The outcomes of this study can be used to design more optimal stimulation protocols that cause less fatigue. Moreover, the level of fatigue can be quantitatively assessed with indicators without the participant’s self-reports.

## 1 Introduction

Brain-computer interface (BCI) system directly connects people’s brains and computers (Heo et al., 2017; Liu et al., 2020). This technology enables healthy and paralyzed people to control external equipment by analyzing brain activity (Shu et al., 2019). BCI has become one of the most important and frequently discussed issues in recent years.

**Figure 1:**
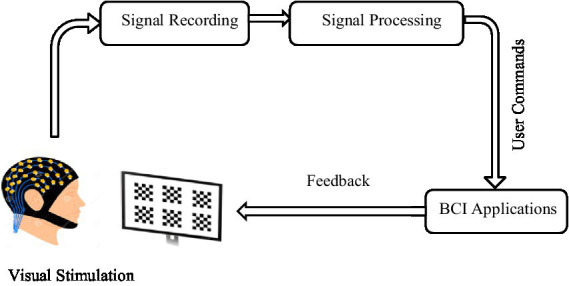
Block diagram of a BCI system.

According to Figure 1, the BCI system analyzes brain signals and converts them into commands to external devices such as a speller, wheelchair, robotic arm, or drone. The target population is patients with severe neuromuscular disorders such as severe paralysis due to muscle wasting, Amyotrophic Lateral Sclerosis (ALS) with the ability to reliably control eye gaze, or brain stem stroke (Demir et al., 2019). However, advanced BCI systems help people by providing an alternative way to communicate, control, and security (Demir et al., 2019).

Electrophysiological/magnetophysiological methods like electroencephalography (EEG), electrocorticography (ECoG), and magnetoencephalography (MEG) are typically used in BCI systems. EEG is the most commonly used modality in BCI systems because of its portability, high temporal resolution, simplicity, and low cost (Collura, 2002).

Six distinct brain rhythms can be distinguished in the EEG signal based on frequency ranges: delta (0.5–4 Hz), theta (4–8 Hz), alpha (8–13 Hz), mu (8–13 Hz), beta (13–30 Hz), and gamma (25–100 Hz). The delta rhythm occurs when toddlers or adults fall asleep deeply or in people with brain abnormalities, comprises frequency components below 3.5 Hz. Theta rhythm occurs When a person is fatigued and unable to concentrate, predominantly appearing in the temporal and parietal areas. The occipital lobes are used to record the alpha rhythm. When individuals are asleep, this rhythm completely disappears, but when they are calm and awake, sleepy but awake, and fatigued, it appears. Also, if the individual tries to stay conscious, alpha will dominate. The beta rhythm is mainly generated in the parietal and frontal areas. Beta happens when a person is attentive, aroused, or excited (Brismar, 2007; Miller, 2007; Foong et al., 2019). The mu-rhythm and gamma rhythm can be recorded from the sensory-motor regions and the somatosensory cortex, respectively. Gamma rhythm is crucial in learning, memorizing, and processing data. Also, it can be seen in high-level cognitive tasks (Herrmann and Demiralp, 2005; Fazel-Rezai et al., 2013).

Steady-state visual evoked potential (SSVEP) is used alongside slow cortical potential (SCP), P300, and event-related synchronization (ERS)/event-related desynchronization (ERD) in brain signal-based BCI systems. A stimulus with frequencies higher than approximately 4 Hz causes SSVEP, a specific type of VEP (Martínez et al., 2010). SSVEP-based BCI systems typically have several benefits over alternative methods, including a higher information transfer rate (ITR) (Sadeghi and Maleki, 2019), better classification accuracy, and fewer recording electrodes. These systems also require less training and have higher user and patient validity (Sadeghi and Maleki, 2018; Maleki and Azadimoghadam, 2022; Deng et al., 2023).

**Figure 2:**
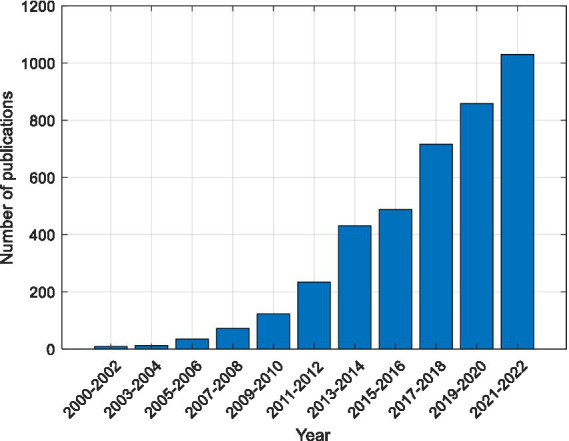
Distribution of published work related to Fatigue in SSVEP-based BCI. These articles were extracted using Google Scholar with the keywords “Fatigue” and “SSVEP”.

Due to its benefits, SSVEP has generated interest in several real-world applications, including operating robots (Kawase et al., 2017), spelling (Kundu and Ari, 2021; Li et al., 2021), gaming (Liao et al., 2012), and driving (Wu et al., 2022). However, fatigue is a major challenge in the practical use of SSVEP (Cao et al., 2014). Numerous efforts have been made in recent years to determine the level of fatigue in BCI systems and to reduce it. The number of publications on Fatigue in SSVEP has increased rapidly in recent years, as shown in Figure 2.

The National Institute of Health defines fatigue as tiredness, a feeling of weariness, or lack of energy. Some factors that influence the process of getting fatigued are time and state of sleep, physical condition, work environment, and work conditions like noise or temperature.

Fatigue brings about both physical and psychological manifestations. On a physical level, it leads to signs like lethargy, insomnia, and headaches. Psychologically, it also influences people’s emotions and behavior. The sensation of apathy and sluggishness illustrates how fatigue influences one’s emotional state, while a decrease in energy highlights its impact on behavioral patterns (Mock-Mclaughlin, 2004).

In the research conducted on SSVEP-based BCI fatigue, two phrases, namely “Mental fatigue” and “Visual fatigue,” have been employed. In the following, we will explore these individual terms. Mental fatigue is a feeling that a person experiences during the period of cognitive activity or after it, such as long-term study and continuous attention to a task. Anxiety and worry can also cause mental fatigue. For people experiencing mental fatigue, it would be difficult to pay attention to something they are not interested in, but it is easy to pay attention to something they enjoy. Generally, there are two types of attention: Involuntary attention and voluntary attention. Involuntary attention is associated to activities that captivate and thrill an individual, requiring no conscious effort to sustain focus. On the other hand, voluntary attention demands a deliberate exertion to stay attentive. Consequently, deliberately directing attention can lead to mental fatigue (Taylor, 2008; Landau et al., 2012). Mental fatigue appears as sleepiness, lethargy or indifference to tasks and reduced subject performance (Marcora et al., 2009). Visual fatigue is exacerbated by many of the same factors that cause general fatigue. Excessive pressure on the retina, constantly switching between intraocular muscles (sphincter, ciliary muscle, dilator papillae, papillae) and extraocular muscles (superior rectus, medial rectus, lateral rectus, inferior rectus, superior oblique, inferior oblique) to get a clear image, and increased intraocular pressure from frowning or neurohormonal action all contribute to visual fatigue (Kels et al., 2015).

In previous studies, the recording protocols have been designed in two ways: continuous and divided. In the continuous approach, the participant consistently gazes at the stimuli for extended periods without any intervals for breaks. In contrast, within the divided protocol, the stimulus flickers during each trial, accompanied by short breaks between trials. These experiments elicit excitement initially, capturing the subject’s focus on the task. However, as the experiment goes on, the task becomes monotonous, leading the participant to lose both interest and focus, eventually leading to decreased performance. Consequently, before commencing the experiment, participants are directed to concentrate exclusively on the target stimulation. This act of attentive focus is voluntary and gradually contributes to mental fatigue over time.

Fatigue is usually detected using a questionnaire (Lewis and Wessely, 1992), and participants are asked about their condition (Lee et al., 1991; Chalder et al., 1993). Tests such as the psychomotor vigilance test (PVT) are also used to assess fatigue (Basner and Rubinstein, 2011; Tan et al., 2013). Several other methods, including EEG signal (Fan et al., 2015; Hou et al., 2015), skin conductance response (SCR) (Bundele and Banerjee, 2009), heart rate monitoring, and oxygen intake (Bundele and Banerjee, 2009), have been used to measure fatigue in recent studies in addition to questionnaires. EEG signals are preferred to other modalities due to their higher temporal resolution (Collura, 2002).

Recently, SSVEP-based BCIs have been employed in the diagnosis of color vision, hand prostheses and wheelchair control, speller, browsing the web, and computer games in the real world, where fatigue is the main challenge. Prolonged use of cues with different patterns, colors and brightness to diagnose of color vision led to visual fatigue (Zheng et al., 2021). Another application is controlling a hand prosthesis to rotate, close and open (Müller-Putz and Pfurtscheller, 2008), and controlling a wheelchair to turn right or left, move forward and stop (Müller et al., 2011). Prolonged use of the wheelchair and hand prosthesis can lead to visual fatigue and paying attention to the target frequency to perform commands leads to mental fatigue. In the application of spelling and web browsing, a virtual keyboard is used, which makes it necessary to focus and pay attention to the order of the letters in the word, to remember their position and the stimulation cue. Also, playing games requires players to maintain high levels of attention in order to react quickly (Parafita et al., 2013). In spelling, web browsing and playing, users initially experience mental fatigue due to the need for concentration to transmit information. If they use the system for a long time, visual fatigue may occur (Lin et al., 2019; Mannan et al., 2020). Therefore, fatigue is a serious challenge when applying an SSVEP-based BCI from the laboratory to the real world. Numerous efforts have been made in recent studies to determine the level of fatigue using quantitative biological indices and to reduce it. Considering the variety and breadth of research on fatigue, and the contradictions and inconsistencies of fatigue indices, it is clear that a systematic review and meta-analysis are necessary. A systematic review endeavors to aggregate all accessible empirical evidence through well-defined and systematic methods. Meanwhile, a meta-analysis involves employing statistical techniques to collate and analyze data from numerous interconnected studies. This paper focuses on the following questions.

How does the stimulation pattern affect fatigue?
What indicators were used to assess fatigue?
Which of the quantitative indicators is more valid for evaluating fatigue?

Continuing onward, the process of selecting research for a systematic review is explained. Subsequently, factors influencing user fatigue are presented. Quantitative indices for assessing fatigue levels are articulated. Then, the quantitative outcomes of prior studies are statistically synthesized through meta-analysis. Finally, a discussion and conclusion are provided.

## 2 Methodology

### 2.1 Search strategy

The present study is a systematic review. Relevant articles were acquired on PubMed, IEEE, and Google Scholar from 2000 to 2022 as the source base for the literature collection. The keywords “SSVEP” AND “Fatigue” and “Steady State Visual Evoked Potential” AND “fatigue” were utilized to search as comprehensively as possible.

### 2.2 Selection criteria

The following criteria were used to select articles for inclusion in the review: (I) The purpose of the articles is to study mental and visual fatigue; (II) The EEG signal resulting from performing a visual task was used as a physiological signal; (III) Quantitative biological criteria and indicators are used to check participant fatigue.

The searched articles were also excluded based on the following criteria: (I) Studies that examined Fatigue in BCI systems based on signals other than EEG and non-visual tasks; (II) Studies that examined mental fatigue based on qualitative indicators.

**Figure 3:**
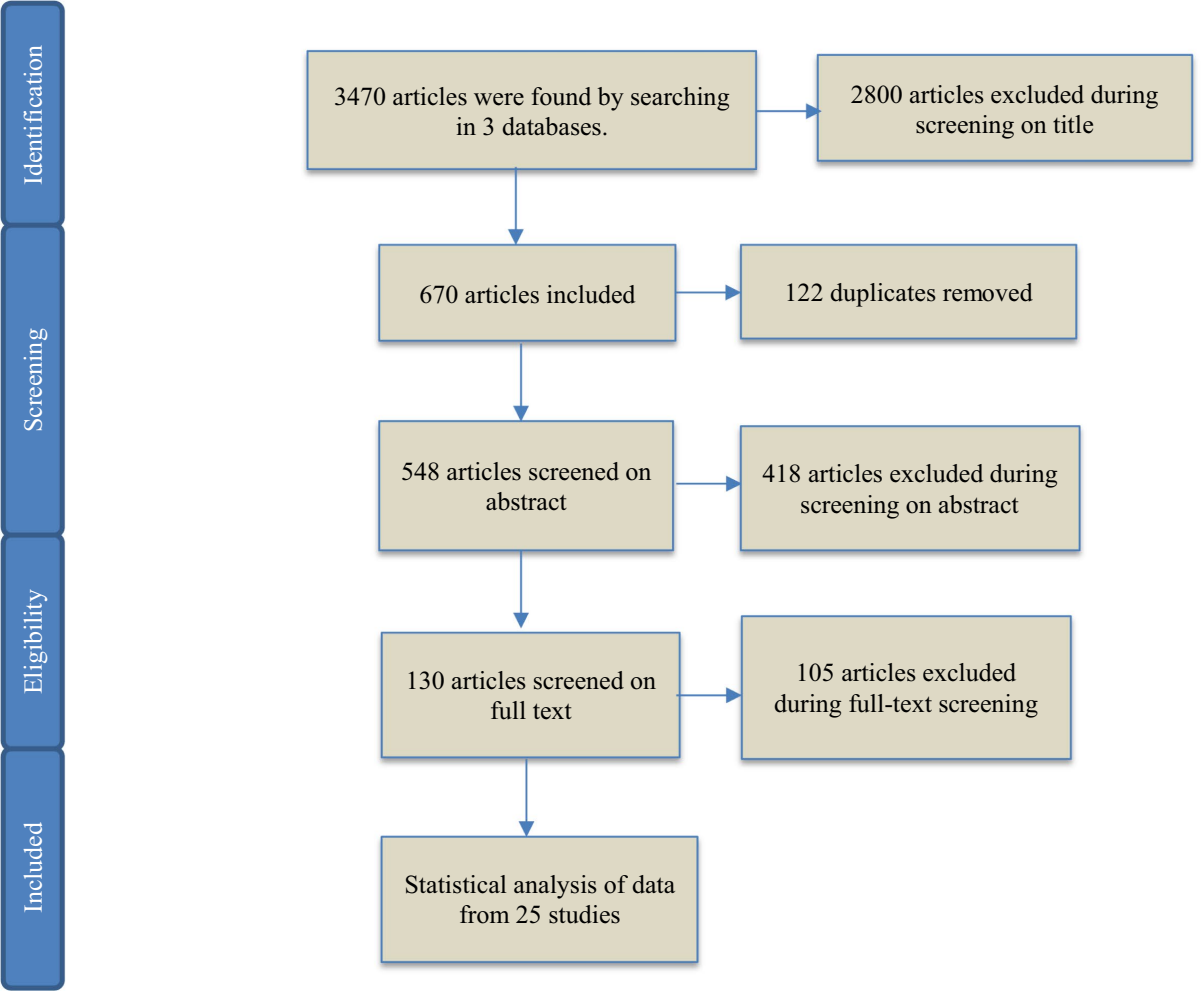
PRISMA flow diagram of studies’ screening and selection.

The full text of 130 articles whose titles and abstracts matched the inclusion and exclusion criteria were evaluated. They are ineligible if, after reading the whole text of the articles, the fatigue was not assessed or if a quantitative indicator was not utilized in the assessment of fatigue, and the distinction between the participant’s alert and fatigue condition was not assessed. 25 articles were ultimately chosen. Figure 3 depicts the entire assessment and selection process for studies. Table 1 shows the characteristics of SSVEP papers.

**Table 1:** Characteristics of papers investigating mental fatigue in SSVEP-based BCIs.

Number	Paper	Number of Subjects	Stimulation Frequency (HZ)	Number of Classes	Channels	Distance (cm)	Inter-Trial Interval (s)	Stimuli Durations (s)	Subjects’ Age	Subject’s Conditions	Stimulation’s Design	Cue’s Color	Objective Evaluation	Subjective Index
1	Chai et al. (2019)	8	8, 9, 10, 11, 12, 13, 14, 15	8	8 channels (PO8, PO4, PO7, PO3, POz, O1, Oz, and O2)	70	1.5	4	22–27 years old.	Normal visual acuity	Motion	Color ranges from white to black	Accuracy, ITR	Custom questionnaire (Bieger and Molina, 2010)
2	Zheng et al. (2020)	12	7.5	1	6 channels (PO3, PO4, POz, O1, O2, and Oz)	60	0.5	5	21–25 years old.	Normal visual acuity	Motion	Color ranges from white to black	SNR, SSVEP amplitude, α+θ	NASA-TLX
3	Seo et al. (2019)	54	12, 8.57, 6.67, 5.45	4	62 channels	60	4	4	24–35 years old.	Normal visual acuity	Flicker	white	α, signal quality	Likert Scale
4	Xie et al. (2016)	9	8, 12, 13.33, 15	4	1 channel (Oz)	70	5	5	23–29 years old.	Normal visual acuity	Motion	Color ranges from white to black	α, θ, θ+α,θ/α, SSVEP amplitude, SNR	…
5	Dang et al. (2021)	8	6.67, 7.5, 8.75, 10, 12	5	8 channels (T5, P3, Pz, P4, T6, O1, Oz, and O2)	…	2	3	…	Normal visual acuity	Motion	Color ranges from white to black	Accuracy	Chalder Fatigue Scale (CFS) (Chalder et al., 1993)
6	Peng et al. (2021)	11	8, 10, 12, 15, 20, 30	6	1 channel (Oz)	50	3	3	21–29 years old.	Normal visual acuity	Flicker	white	α, β, θ, δ, $\frac{\alpha +\theta }{\beta }$, α/β, θ/β, θ/α θ+α, θ+α+β, SNR, sample entropy	Chalder Fatigue Scale (CFS)
7	Peng et al. (2019)	12	8, 10, 12, 15, 20, 30	6	1 channel (Oz)	50	3	3	21–29 years old.	Normal visual acuity	Flicker	white	α, β, θ, δ, $\frac{\alpha +\theta }{\beta }$, α/β, θ/β, θ/α θ+α, θ+α+β, SNR, MSE	Chalder Fatigue Scale (CFS)
8	Cao et al. (2014)	8	15	1	1 channel (Oz)	50	2	3	21–29 years old.	Normal visual acuity	Flicker	white	α,$\left(\theta +\alpha \right)/\beta$, θ/α, SSVEP amplitude, SNR	Chalder Fatigue Scale (CFS)
		13	7–12	6										
			13–18	6										
9	Benda et al. (2018)	8	6.32, 7.06, 8, 9.23	4	8 channels (Pz, PO3, PO4, O1, O2, Oz, O9, and O10)	60	…	…	Average 28.13 years old.	Normal visual acuity	Flicker	white	α, β, θ, δ,θ/α, $\frac{\alpha +\theta }{\beta }$	Chalder Fatigue Scale (CFS)
10	Keihani et al. (2018)	22	25, 30, 35	3	3 channels (Oz, O1, and O2)	75	2	6	23–30 years old.	Normal visual acuity	Flicker	White LED	Accuracy	Visual Analogue Scale (VAS) (Shahid et al., 2011)
11	Makri et al. (2015)	13	6.67, 7.5, 8.57, 10	4	4 channels (O1, O2, Oz and POz)	80	90	150	22–39 years old.	Normal visual acuity	Flicker	Checkerboard white and black, white square	α, β, θ, δ-θ/α, $\frac{\alpha +\theta }{\beta }$, SSVEP amplitude	Chalder Fatigue Scale (CFS)
12	Palaniappan et al. (2018)	5	7	1	14 channels	…	…	…	25–26 years old.	Normal visual acuity	Flicker	…	β	…
13	Lee et al. (2018)	10	5.45, 6.67, 8.57	3	19 channels	50	5	6	24–32 years old.	Normal visual acuity	Flicker	white	band power at the target frequency, accuracy	simple questionnaire
14	Demir et al. (2019)	4	8,14, 28	3	128 channels	90	5	15	…	Normal visual acuity	Flicker	black and white checkerboards	PSDA, CCA, UF, BIFB	…
		4	6, 6.5, 7, 7.5, 8.2, 9.3, 10	7	3 channels			30						
15	Chai et al. (2020)	14	8–15	8	8 channels (PO8, PO4, PO7, PO8, POz, O1, Oz, and O2)	70	3.5	5	Average 24.9 years old.	Normal visual acuity	Flicker and Motion	Blue, Green, Gray, White	Accuracy, SNR, $\frac{\alpha }{\theta }$	…
			38–45	8										
			8–12.8	8										

Since in addition to the SSVEP-based BCI systems, visual task fatigue occurs in driving studies and prolonged 3D TV watching, research in these areas is also considered. It is important to note that although SSVEP-based BCI systems and driving/long-time 3D TV watching are distinct applications, the signal processing approach to evaluate fatigue in these applications is similar. Hence, the results of research in these two fields can be used to evaluate fatigue in BCI systems based on SSVEP. Table 2 shows the characteristics of papers investigating mental fatigue in driving and 3D TV using EEG signals.

**Table 2:** Characteristics of papers investigating mental fatigue in driving and 3D TV using EEG signals.

Number	Paper	Number of Subjects	Type of the Task	Channels	Distance (m)	Inter-trial Interval (min)	Stimuli Durations (min)	Subjects’ Age	Subject’s Conditions	Objective Evaluation	Subjective Index
1	Chen et al. (2013)	10	3D TV	16 channels	3	…	40	20–24 years old.	normal visual acuity	β, β, θ, δ, $\frac{\alpha +\theta }{\beta }$, α/β, θ/β, $\left(\alpha +\theta \right)/\left(\alpha +\beta \right)$	Subjective questionnaire
2	Guo et al. (2018)	66	Driving	64 channels	…	…	…	Average 23.25 years old.	normal visual acuity	percentage of eye closure-standard deviation of the lane position-Profile of Mood States-	POMS-SF (Chen et al., 2002)
3	Lal and Craig (2005)	35	Driving	19 channels	…	10	10–15	Average 34	normal visual acuity	α, β, θ, δ	…
		20						Average 44			
4	Jap et al. (2009)	52	Driving	30 channels	…	…	10–15	Average 28 years	normal visual acuity	α, β, θ, δ,$\frac{\alpha +\theta }{\beta }$, α/β, $\left(\alpha +\theta \right)/\left(\alpha +\beta \right)$, θ/β	Lifestyle appraisal questionnaire (Craig et al., 1996)
5	Zou et al. (2015)	11	3D TV	30 channels	…	…	10	Average 26.08 years	normal visual acuity	α, β, θ,$\frac{\alpha +\theta }{\beta }$, α/β, $\left(\alpha +\theta \right)/\left(\alpha +\beta \right)$, θ/β α/θ, $\theta /\left(\alpha +\beta \right)$	Sheedy’s questionnaire
6	Bang et al. (2014)	15	3D TV	16 channels	2.5	5	30	Average 26.89 years	normal visual acuity	β	custom questionnaire
7	Eoh et al. (2005)	4	Driving	8 channels (FP1, FP2, T3, T4, P3, P4, O1, and O2)	…	…	…	Average 26.1 years	normal visual acuity	α, β, θ, θ/α,β/α, $\left(\theta +\alpha \right)/\beta$	…
8	Wang et al. (2022)	10	Driving	2 channels (C3, C4)	…	30	120	Average 24 years	normal visual acuity	mass exponent, Hurst exponent, multifractal spectrum of θ and β	Subjective questionnaire
9	Sheykhivand et al. (2022)	11	Driving	2 channels (FC3, FCz)	…	…	20	22–30 years old.	normal visual acuity	Accuracy	Chalder fatigue, Lee fatigue scales
10	Wang et al. (2022)	10	Driving	14 channels			180	24∓0.3 years old.	normal visual acuity	basic scale entropy of α and β band	Subjective questionnaire

### 2.3 Meta-analysis of quantitative results of studies

The statistical technique for combining the results of independent studies is called a meta-analysis (Gurevitch and Hedges, 2020). To use this method, the articles that included the exclusion criteria were left out of the meta-analysis. These criteria include:

1. Studies whose characteristic value is not quantified (represented by a number).
2. Studies in which the results’ standard deviation is unknown.
3. Studies in which the quantitative value of the feature cannot distinguish between states of alertness and fatigue.

Also, if the desired index was used in more than three studies, it was included in the meta-analysis method. Then, the mean and standard deviation of indices such as frequency recognition accuracy, signal-to-noise ratio (SNR), SSVEP amplitude, alpha, beta, theta, delta frequency bands, and power ratio $\frac{\alpha +\theta }{\beta }$ with their sample size were extracted. In articles where the results were not presented quantitatively, indices were extracted from graphs using “graphreader.com.”

Comprehensive Meta-Analysis V3 (CMA) software was used to perform meta-analysis statistical calculations. Using CMA software, the z-test has been used to evaluate the null hypothesis of the effect size, and the magnitude of heterogeneity is estimated by the $I^{2}$ (Dettori et al., 2021).

## 3 Results

### 3.1 Factors affecting fatigue

In SSVEP-based BCI systems, the visual stimulation paradigm and stimulation frequency are effective in fatigue.

#### 3.1.1 Visual stimulation paradigm

Previous studies investigated the level of fatigue caused by various stimulation paradigms. For this purpose, first different visual stimulation patterns are shown to the participants, then using objective and subjective fatigue index, the impact of the paradigm on the fatigue is determined. The characteristics of prolonged repetitive visual stimuli are divided into stimulus cues and background. Stimulus cues are visual stimuli displayed on the screen with a certain frequency. These cues can be repeated in two ways: Flicker and Motion. Flickering is the repeated presentation of two colors of cue for durations $T_{1}$ and $T_{2}$, leading to the SSVEP response. The value of duty-cycle, determined by the ratio of $T_{1}$ to $T_{1}+T_{2}$, can affect the fatigue caused by flickering stimulus (Craig et al., 1996; Eoh et al., 2005). Motion refers to the condition in which the cue is moving and this movement leads to visual stimulation. This process gives rise to the responses known as steady-state motion visual evoked potentials (SSMVEP).

The visual stimulation cues are displayed on the screen with different sizes and shapes, such as circles and squares (Xie et al., 2016; Chai et al., 2019). Various patterns are used to display these cues such as single graphic (Chai et al., 2019), grating (Zheng et al., 2020), and checkerboards (Zheng et al., 2020). Cues can also be created using different colors. Typically, black and white are utilized for displays, but green, blue, and gray were employed to investigate the effect of color on fatigue (Xie et al., 2016).

**Figure 4:**
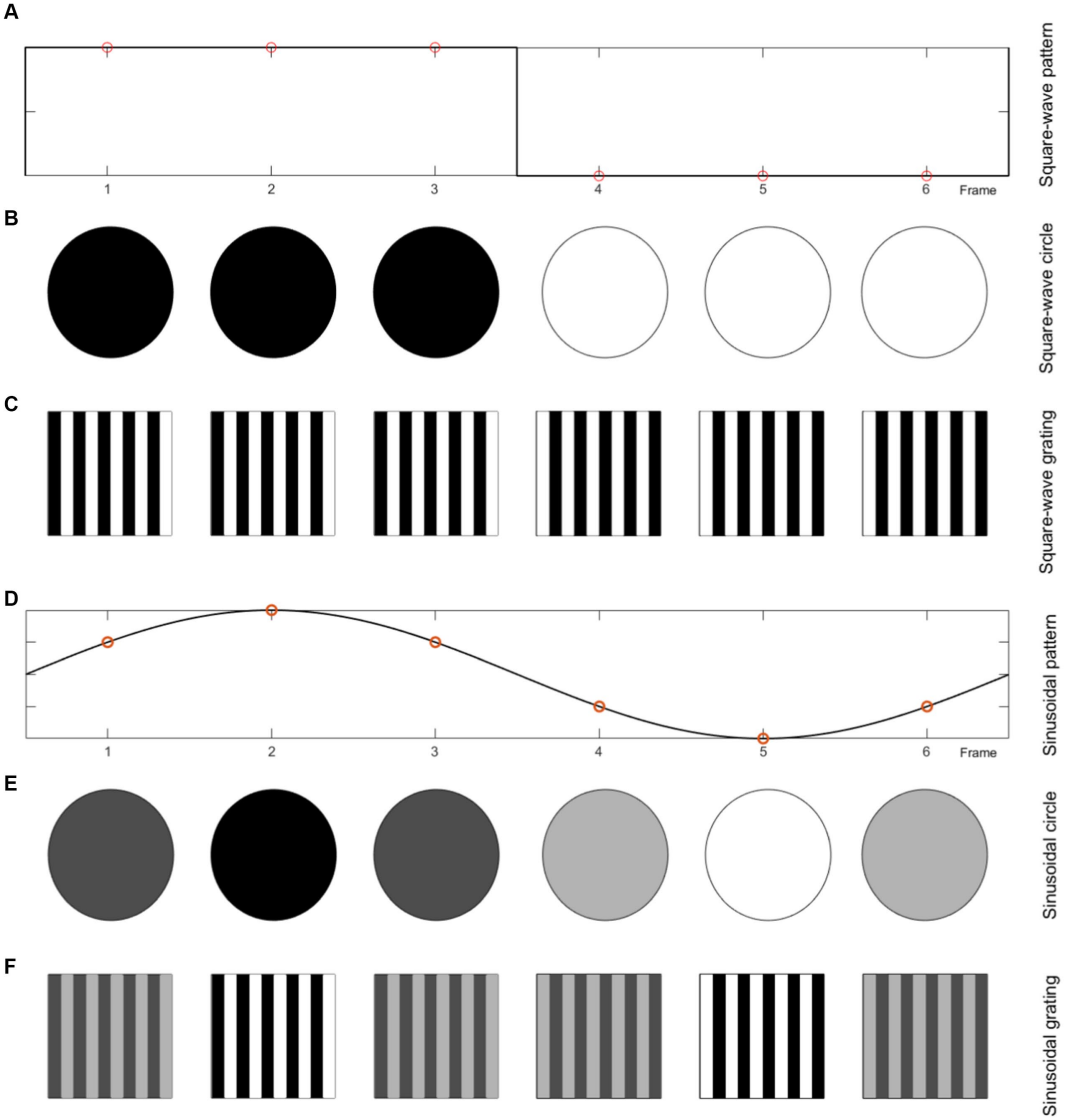
Flicker, each frame of the 10 Hz stimulation with 60 Hz display refresh rate for different paradigms. **(A)** Square-wave pattern. **(B)** Circle (single graphic) with square-wave color intensity change. **(C)** Grating pattern with square-wave color intensity change. **(D)** Sinusoidal pattern. **(E)** Circle (single graphic) with sinusoidal color intensity change. **(F)** Grating pattern with sinusoidal color intensity change.

**Figure 5:**
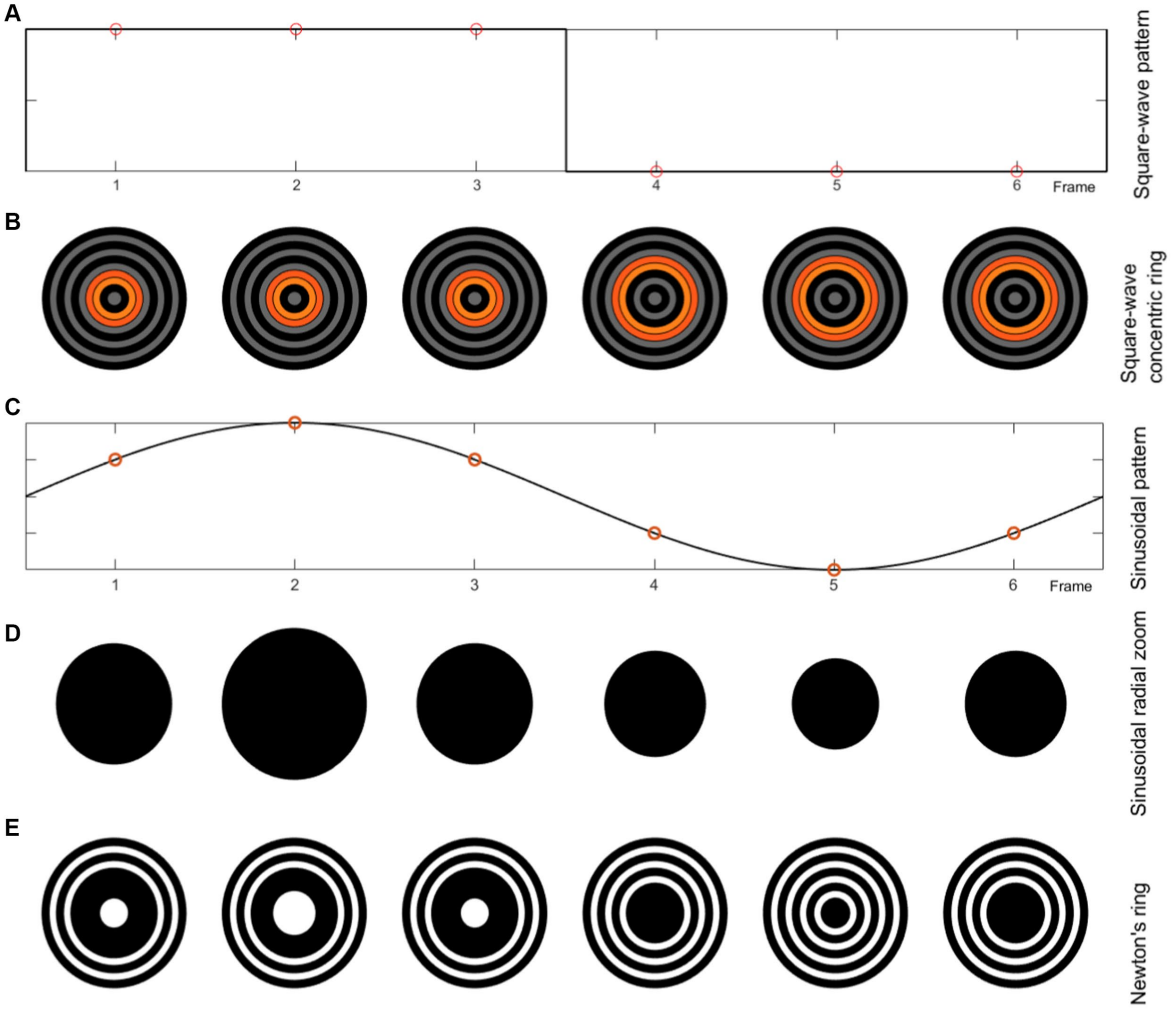
Motion mode, each frame of the 10 Hz stimulation with 60 HZ display refresh rate for different paradigms. **(A)** Square-wave pattern. **(B)** Concentric ring with square-wave change pattern. **(C)** Sinusoidal pattern. **(D)** Sinusoidal radial zoom motion of a circle (single graphic) with the same color intensity. **(E)** Newton’s ring with sinusoidal change pattern.

The color intensity mode and cues motion modes can be created with square and sinusoidal modulation. Figure 4 shows the stimuli generation procedure for one period of flicker mode with changing color intensity with square-wave and sinusoidal patterns using circle and square shapes, and single graphic and grating pattern. Figure 5 shows the stimuli generation procedure in one period of motion mode with changing color intensity with square-wave and sinosoidal patterns using concentric ring, radial zoom motion and Newton’s ring.

As described, another characteristic of prolonged repetitive visual stimuli is background. Usually, a black screen is used as a background for visual stimulation (Xie et al., 2016; Chai et al., 2019; Zheng et al., 2020). However, in Palaniappan et al. (2018), the effect of a black screen, a video, and white noise as a background on fatigue has been investigated.

#### 3.1.2 Stimulation frequencies

According to BCI studies, stimulation frequencies are divided into three categories: low frequency (1 to 12 Hz), medium frequency (12 to 30 Hz), and high frequency (30 to 50 Hz) (Zhu et al., 2010; Volosyak et al., 2011; Chabuda et al., 2017; Keihani et al., 2018). Most studies on SSVEP have used the low and medium frequency band, which includes frequencies from 5 to 25 Hz. It has been shown in Xie et al. (2016) and Diez et al. (2011) that high stimulation frequency leads to less subject fatigue. In other words, different stimulation frequencies can induce different levels of fatigue (Diez et al., 2011).

### 3.2 Fatigue quantification

**Figure 6:**
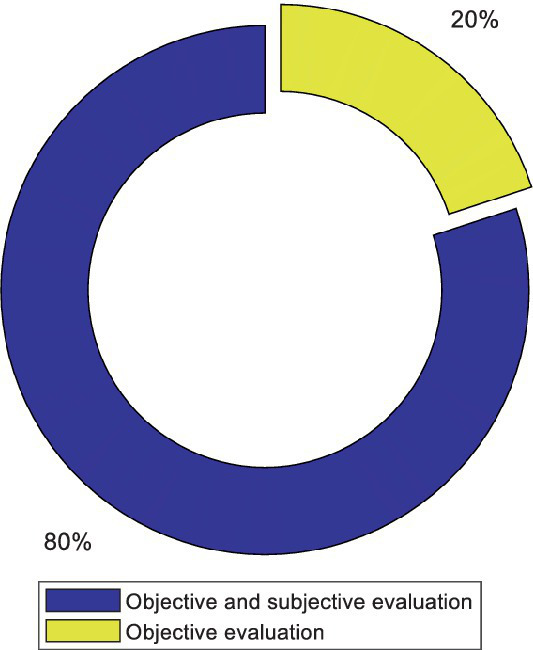
Comparison of the number of articles that used only the objective evaluation method and the articles that used the subjective and objective methods simultaneously to assess fatigue.

Previous studies have employed two objective and/or subjective evaluation techniques before and after each experiment to assess fatigue induced by visual stimulation. Subjective evaluation involves assessing the subject’s performance and state through a questionnaire that is provided to the subject. Objective evaluation analyzes the data using quantitative indicators to determine the subject’s state, including alertness and fatigue. Figure 6 shows the percentage of studies that used both objective and subjective evaluations to investigate fatigue, compared to studies that used only objective evaluation. In most studies, both methods have been used to evaluate fatigue.

#### 3.2.1 Subjective evaluation

In recent studies, the NASA-TLX and Chalder questionnaires are the most frequently utilized fatigue questionnaires. In the 1980s, researchers at NASA Ames Research Center designed the NASA-TLX as a paper-and-pencil questionnaire. The NASA-TLX has established the standard for evaluating subjective workload across various applications (Storer, 2019). This method has been used in many studies as a measurement tool to assess fatigue. In these studies, it is assumed that the workload inferred by NASA-TLX indicates mental fatigue. This questionnaire examines six characteristics of mental demand (MD), physical demand (PD), temporal demand (TD), performance (PE), effort (EF), and frustration (FR). After completing the experiment, participants are asked to rate from 0 to 100. Finally, the results are ranked using the weighted average of the characteristics (Sampei et al., 2016).

In 1993, Chalder et al. developed an instrument of 14 questions to measure fatigue, which measures fatigue’s physical and mental symptoms. All participants were required to complete a self-reported fatigue questionnaire based on the Chalder Fatigue Scale (CFS) before and after the experiment. The CFS had a high level of reliability and validity and consisted of eight physical and six mental fatigue items. Every question had four choices rated on a four-point scale (0–3). A high fatigue score presented a high level of fatigue (Chalder et al., 1993).

There are some serious limitations to the questionnaire. Subjects may have recognized the researchers’ intention behind the questions and provided answers appropriately. Furthermore, subjective fatigue ratings are unsuitable for online BCI applications since it is impractical to expect a BCI user to respond to a frequent inquiry (Li et al., 2008). Therefore, subjective fatigue assessments may be unpleasant or unworkable for use in BCI fatigue measurement applications. The fatigue indices based on physiologic signals seem more objective and appropriate for BCI applications than subjective fatigue evaluations. According to Figure 6, methods based on physiologic signals have also been used in addition to an objective evaluation in many studies.

#### 3.2.2 Objective evaluation

**Figure 7:**
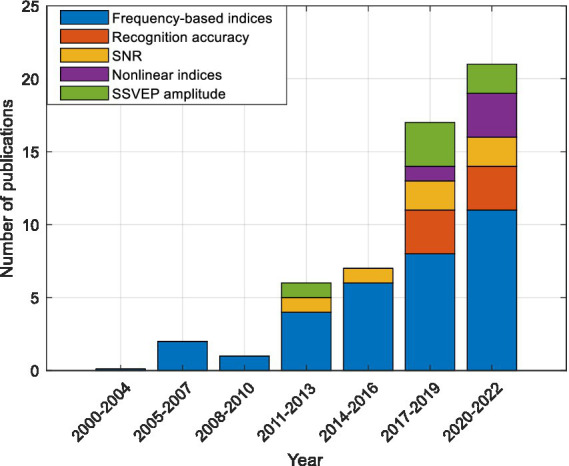
The number of articles published in the field of fatigue detection using frequency-based indicators, recognition accuracy, SNR, SSVEP amplitude, and nonlinear indices.

In previous studies, various indices such as frequency-based indices, frequency recognition accuracy, SNR, SSVEP amplitude, and nonlinear indices have been used to investigate the fatigue level of participants. The number of articles published regarding each fatigue index from 2000 to 2022 is shown in Figure 7.

According to Figure 7, in previous studies, the frequency indices have most frequently been used to evaluate fatigue between the alert and fatigue states. These indices have been proposed since 2005 and are used until today. The ranges of delta, theta, alpha and beta frequency bands were chosen as 0.5–4 Hz, 4–8 Hz, 8–13 Hz, and 13–30 Hz, respectively.

The ratio of the EEG amplitude at stimulus frequency (SSVEP signal) to the EEG amplitude at surrounding frequencies (noise) is considered SSVEP SNR, as described by


(1) $$SNR=\frac{\sum \left|Z\left(f_{t}\mp \Delta \;f_{s}\right)\right|}{\sum \left|Z\left(f_{t}\mp \Delta \;f_{n}\right)\right|-\sum \left|Z\left(f_{t}\mp \Delta \;f_{s}\right)\right|}$$

$Z\left(f\right)$ is the SSVEP spectrum calculated by fast Fourier transform (FFT), $f_{t}$ is the stimulus frequency, $\Delta \;f_{s}$ is the frequency range of signal and $\Delta \;f_{n}$ is the frequency range of noise (Ziafati and Maleki, 2022). Previous studies have shown that when the individual becomes fatigued, the SNR value decreases. The SSVEP amplitude is also an index used to evaluate fatigue in various studies, and its value decreases when the subject is fatigued.

Other indicators such as frequency recognition accuracy and nonlinear indices have been recently used to determine the subject’s fatigue level. Power spectral density analysis (PSDA), canonical correlation analysis (CCA), least absolute shrinkage and selection operator analysis (LASSO) methods are used for frequency recognition (Keihani et al., 2018; Chai et al., 2019; Demir et al., 2019).

Multiscale entropy (MSE) is an index that was first used in 2019 to investigate fatigue based on the SSVEP signal (Peng et al., 2019). This feature determines the complexity of the signal and detects fatigue using sample entropy and coarse-grained process. The results have shown that this index is related to the subject’s fatigue and decreases when the participant becomes fatigued.

**Figure 8:**
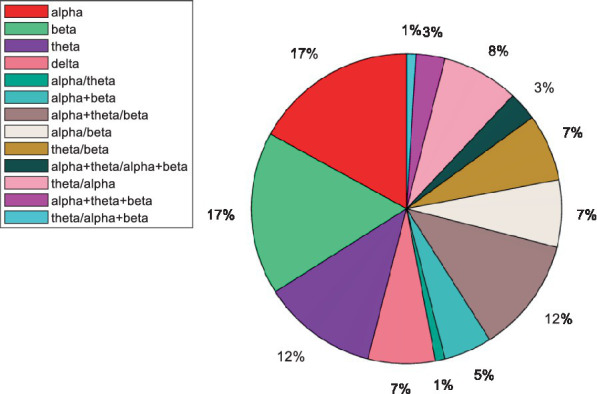
Distribution of frequency indices in articles published from 2000 to 2022.

Figure 8 displays the distribution of the frequency indices in papers published from 2000 to 2022. The alpha and beta frequency bands have been the most used index in past studies. The alpha frequency band has been utilized as an indicator in 17% of the articles independently and in 42% of the articles in combination with other frequency indicators. The combination includes addition and division operators. Also, the beta frequency band has been utilized as an indicator in 17% of the articles independently and in 39% of the articles in combination with other frequency indicators. According to the results, alpha and beta frequency bands have historically been the most frequently used as independent features; however, the use of combination features is more common and more accurately depicts fatigue than the independent mode. Table 3 shows different equations for the estimation of frequency indices.

**Table 3:** Different equations for estimation of frequency indices in previous studies.

Studies	Equation
Peng et al. (2021)	$\text{Normalized}\;\text{sum}\;\text{of}\;\text{spectrum}\;\text{amplitudes}=\frac{\sum_{f=f_{1}}^{f_{2}}\left|Z\left(f\right)\right|}{\sum_{f=4}^{30}\left|Z\left(f\right)\right|}$
Benda et al. (2018) and Cao et al. (2014)	$\text{Mean}\;\text{of}\;\text{spectrum}\;\text{amplitudes}=\frac{\sum_{f_{1}}^{f_{2}}\left|Z\left(f\right)\right|}{N}$
Zheng et al. (2020) and Jap et al. (2009)	$\text{Sum}\;\text{of}\;\text{spectrum}\;\text{amplitudes}=\sum_{f=f_{1}}^{f_{2}}\left|Z\left(f\right)\right|$
Chen et al. (2013)	$\text{Sum}\;\text{of}\;\text{squered}\;\text{of}\;\text{spectrum}\;\text{amplitudes}=\sum_{f=f_{1}}^{f_{2}}{\left|Z\left(f\right)\right|}^{2}$

$f_{1}$ and $f_{2}$ show the frequency range of different frequency bands and N is the number of frequency points in the desired frequency band. $Z\left(f\right)$ is the frequency spectrum of the SSVEP signal calculated using FFT, and $\left|Z\left(f\right)\right|$ is the amplitude of the frequency spectrum.

### 3.3 Meta-analysis of fatigue indices

**Figure 9:**
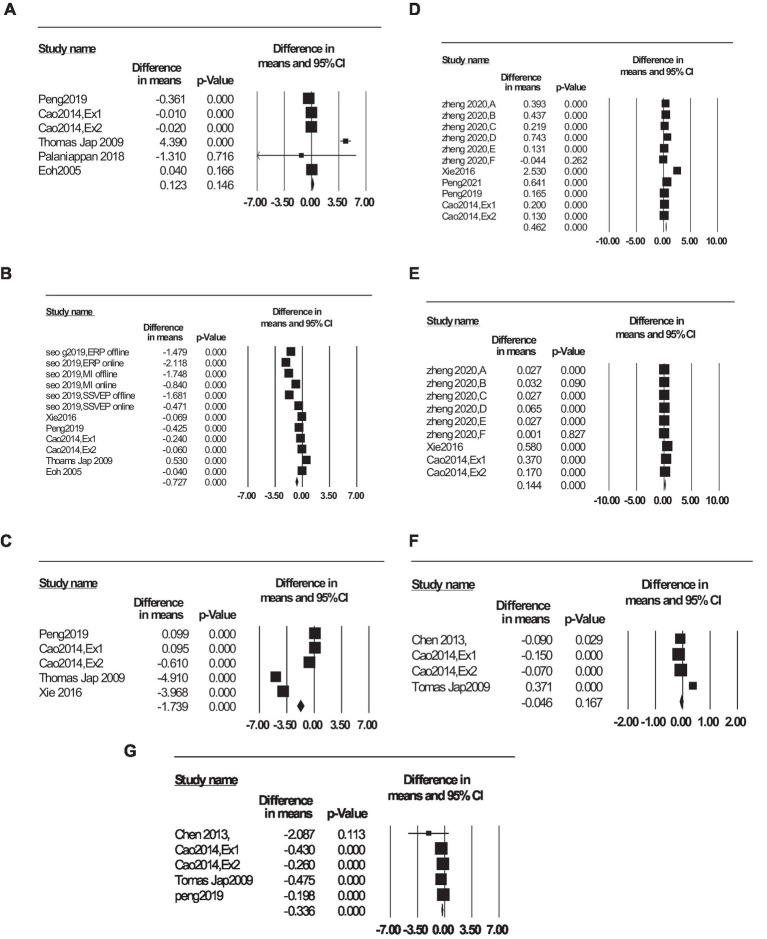
Forest plot of fatigue indices: **(A)** β, **(B)** α, **(C)** θ, **(D)** SNR, **(E)** SSVEP amp, **(F)** δ, and **(G)** $\frac{\alpha +\theta }{\beta }$. Squares represent each study’s effect size estimates, and horizontal lines represent confidence intervals. Diamonds represent point estimates and confidence intervals after combining and averaging all the studies together.

Different fatigue indices have been investigated by meta-analysis and the results including effect size, confidence interval, value of *p*, and $I^{2}$ are shown in Table 4. Effect size is a simple way to quantify the difference between two groups. A value of *p*, or probability value, describes how likely the data would have occurred under the null hypothesis of a statistical test. The $I^{2}$ statistic describes the percentage of variation across studies that is due to heterogeneity. The high percentage of $I^{2}$ can be caused by different measurement methods and tools to calculate the characteristics, clinical characteristics, and differences between subjects. Forest plots are presented in Figure 9.

**Table 4:** Meta-analysis results.

Group	Effect size (Difference in means)	Confidence interval		*p* value	$I^{2}$
		Upper limit	Lower limit		
α	−0.727	−0.573	−0.881	< 0.001	99.994
β	0.123	0.288	−0.043	0.146	99.995
θ	−1.739	−1.289	−2.188	< 0.001	99.993
δ	−0.046	0.019	−0.112	0.167	99.413
SNR	0.462	0.512	0.412	< 0.001	99.828
SSVEP amplitude	0.144	0.225	0.064	< 0.001	99.901
$\frac{\alpha +\theta }{\beta }$	−0.336	−0.267	−0.404	< 0.001	98.787

The $I^{2}$ is greater than 75%, indicating considerable heterogeneity. Reasons for heterogeneity could include clinical characteristics and inconsistencies between studies. According to Figure 9B and Table 4, the α index is good at detecting fatigue because the absolute effect size of the difference in means is high. The negative value of the effect size indicates that as the subjects get fatigued, the amplitude of the α increases. For this index, the value of *p* is less than 0.05, so it is statistically significant. When people are asleep, the alpha rhythm completely disappears. Still, when people are calm and awake, when they are sleepy but awake, and when they are fatigued, α rhythm appears. Therefore, the α index is expected to increase as participants become fatigued and can be used for fatigue detection.

Figure 9C and Table 4 shows that due to the negative value of the difference in means and value of *p* of θ, this index increases significantly as the participants become fatigued. The square’s location in Figure 9C indicates the average difference in means in all studies.

Figure 9D and Table 4 show the changes in SNR with fatigue. According to the positive effect size and value of p, the SNR decreases as the subject fatigues. Also, the diamond in Figure 9 has a distance from the no-effect line, which shows the significance of SNR reduction with fatigue. Since the SSVEP amplitude decreases as the participants’ attention and concentration decrease, the SNR value is expected to decrease as the participants become fatigued.

According to Figure 9E and Table 4, SSVEP amplitude decreases with fatigue, and a *p*-value less than 0.05 indicates a significant decrease in SSVEP amplitude. The evoked part of the SSVEP signal decreases with fatigue, so this result is reasonable.

Figure 9G and Table 4 show that the $\frac{\alpha +\theta }{\beta }$ index increases significantly as the participants get fatigued. This index simultaneously examines changes in the alpha, beta, and theta frequency bands. Considering the increase of alpha and theta frequency bands with subject’s fatigue, using them in the numerator of the fraction, made it a more effective indicator in fatigue detection.

According to Figures 9A,F and Table 4, the beta and delta indices do not change significantly between the alert and fatigue states, so they are not recommended for fatigue detection.

## 4 Discussion

In this systematic review, studies that investigated visual and mental fatigue using visual stimuli were considered. For this purpose, the impact of the visual stimulation paradigm on fatigue and quantitative indices of fatigue was investigated.

### 4.1 Visual stimulation paradigm

Attractiveness and variety are the most effective ways to reduce fatigue caused by visual stimulation. Among the different methods of creating attractiveness in the stimulation pattern, zoom motion (Chai et al., 2019), Newton’s ring motion (Xie et al., 2016; Chai et al., 2019; Zheng et al., 2020), and various patterns of cues (Zheng et al., 2020) are more effective in reducing visual and mental fatigue. Newton’s ring motion may cause a feeling of vertigo (Chai et al., 2019) while zoom motion does not. Checkerboard, vertical grating, and horizontal grating are cue patterns that cause less fatigue than single graphic (Zheng et al., 2020).

In addition to motion and cue patterns, other factors of visual stimulus including size, color and change of cue intensity are also effective on fatigue. The motion of the cue can be realized sinusoidally or squarely during the visual stimulation. The sinusoidal motion mode changes the cue sinusoidally, but the square-wave motion mode changes the cue between two states. The size and color of the cue can also reduce fatigue. The results show that green causes less fatigue than gray and blue. However, further research is required on the effect of diversity and brightness of colors, the compatibility of the cue’s color with the background, and the dependence of these factors on gender.

The intensity transition of the cue can be implemented squarely or sinusoidally during the visual stimulation. In square intensity transition mode, the color of the cue changes from one color to another, but in sinusoidal intensity transition mode, the color of the cue varies according to the color spectrum. It is suggested to investigate changing the intensity transition mode with sinusoidal form during a zoom motion with sinusoidal motion simultaneously. In this case, the fatigue caused by the combination of sinusoidal motion mode and sinusoidal intensity transition mode can be measured. There is also evidence that smaller stimuli can reduce fatigue (Chai et al., 2020).

Although the cue patterns are more effective in reducing fatigue than single graphic due to their attractiveness and variety, there is no significant difference between the checkerboard, horizontal grating, and vertical grating (Zheng et al., 2020). There were no significant differences between the square and circle flicker SSVEP comfort scores (Chai et al., 2019). Background variation, such as white noise and video compared to a black screen, reduces the simplicity and repetitiveness of the stimulus, however, no significant differences were found between the different background scenarios in previous studies (Benda et al., 2018).

In previous research, attempts have been made to investigate different stimulation patterns to reduce user fatigue. However, it is suggested that in addition to focusing on reducing fatigue, the accuracy of frequency recognition should also be considered because reduction of fatigue is valuable if the accuracy of frequency recognition and ITR are not diminished.

**Figure 10:**
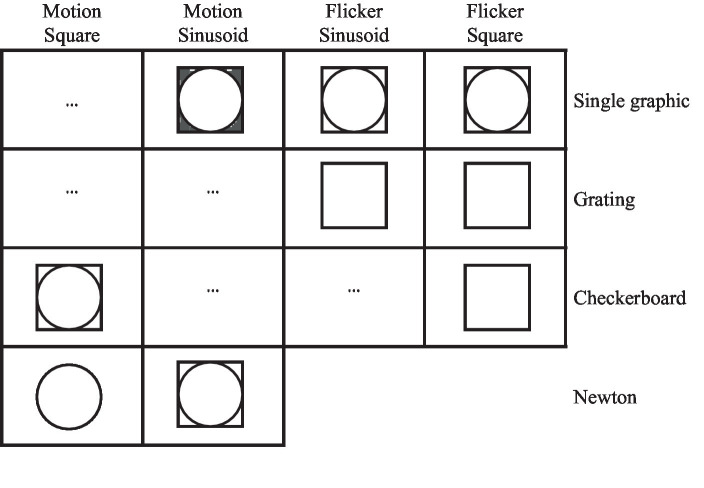
Types of stimulation characteristics used in previous studies. Shape, color, motion/flicker, and pattern create variety in stimulation patterns. 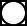 Indicates that both circle and square shapes have been investigated. 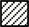 Indicates that different colors were used for stimulation cues.

Mental fatigue refers to the sensation experienced after engaging in prolonged cognitive activities or tasks that require attention and concentration. Visual fatigue occurs when engaging in prolonged visual tasks, employing the eye muscles. The factors of fatigue discussed in this study, such as shape, color, size, cue pattern, and type of motion, can directly impact visual fatigue (Bando et al., 2012) and indirectly affect mental fatigue. Figure 10 displays a collection of stimulation patterns utilized in previous studies by varying the color, shape, pattern, and type of motion. There is no part in Figure 10 dedicated to designing flicker patterns using Newton’s ring because it is impossible to design flicker with Newton’s ring.

### 4.2 Fatigue quantification

Due to questionnaire limitations, it has been attempted to utilize quantitative indicators in addition to the questionnaire in evaluating fatigue. Quantitative indices include frequency indices, SNR, SSVEP signal amplitude, and multiscale entropy. The meta-analysis shows a high percentage of $I^{2}$, indicating the heterogeneity of the results from previous studies.

The utilization of frequency indices has increased in recent years. Initial studies aimed to validate these indices, but subsequently, they have been utilized as an indicator of participant fatigue. The amplitude of the alpha and beta frequency bands are the most prevalent and practical frequency indices used in fatigue detection. The combinations of the alpha frequency band with other frequency bands are the most common combined indices.

The alpha frequency band arises during a mental state characterized by relaxed and effortless alertness, providing a significant indication of either relaxation or low arousal. As the participants become fatigued, their attention and concentration decreases. Hence, to maintain vigilance in the fatigued state, participants must pay more attention to the target and use more mental effort than the alert condition. More mental effort to maintain vigilance in turn increases alpha activity (Cao et al., 2014).

According to the results of previous studies, it has been observed that the frequency indices do not have consistent behavior and are not aligned with the subject’s fatigue. So, it is necessary to use the meta-analysis method. The meta-analysis shows that as the subject gets fatigued, the frequency band amplitude of alpha, theta, and $\frac{\alpha +\theta }{\beta }$ increases significantly. However, no significant change was observed in the amplitude of the beta and delta frequency bands, so it is not recommended to detect the participants’ fatigue. In addition, the meta-analysis results also show that the SNR is significantly affected by users’ fatigue. To compute the SNR index, it is necessary to know the stimulus frequency. Since in practical applications, the stimulation frequency is unknown, this is the main drawback of the SNR (Peng et al., 2019).

How the SNR changes when the subject becomes fatigued is dependent on the stimulation frequency’s location within the SSVEP frequency range. The SNR was defined in past studies as the amplitude ratio at stimulation frequency (signal) to the mean value of the n adjacent points (noise). In this research, regardless of whether the stimulation frequency is in the alpha, beta, or theta frequency bands, the SNR computation and evaluation of how the SNR changes when the subject becomes fatigued were performed. This way of evaluating the effect of fatigue on SNR reveals a disregard for the different responses of various frequency bands to fatigue. Suppose the signal amplitude changes are assumed to be the same for different frequencies. In that case, the meta-analysis results show that the noise (amplitude of different frequency bands) changes differently. Accordingly, it is expected that how SNR changes with fatigue in different frequency bands is different. The entropy has been proposed by Peng et al. (2019) as a fatigue index, which performs well in BCI applications based on SSVEP and does not require stimulation frequency.

The major challenge we encounter is the fatigue index, which evaluates fatigue across multiple trials. Previous research has extensively investigated the realm of fatigue indices within a general context in different BCI applications. As an illustration, the initial 10 trials are considered as the alert, while the final 10 trials are considered as the fatigue. Unfortunately, we lack an index that can investigate fatigue within a single trial or a short time interval. To solve this challenge effectively, it is needed to develop a fatigue index that enables assessment of the fatigue in each trial.

The results reported herein should be considered in the light of some limitations. A larger number of papers would enhance the validity of the results. Considering that the input criteria of the meta-analysis method are the existence of three sources for review, some criteria did not have enough references and were not investigated. In this study, our investigation focused on β, δ, α, θ, SNR, SSVEP amplitude and $\frac{\alpha +\theta }{\beta }$ as we lacked adequate papers to investigate another factor in meta-analysis.

Also, in some studies, the values of fatigue factors in alert and fatigue states were not explicitly provided. This absence prevented us from making comparisons between factors. Consequently, these factors were not within the scope of this study’s investigation.

## 5 Conclusion

In conclusion, this systematic review focused on studies investigating fatigue induced by visual stimuli. The study has delved into the impact of the visual stimulation paradigm on fatigue and quantitative indices to evaluate it.

Regarding the visual stimulation paradigm, it was found that attractiveness and variety are effective ways to alleviate fatigue induced by visual stimulation. Notably, techniques like zoom motion, Newton’s ring motion, and various cue patterns were more effective in reducing fatigue. However, Newton’s ring motion might lead to feelings of vertigo, which is not the case with zoom motion. Additionally, factors like cue size and color played a role in fatigue reduction, with green being less fatiguing than gray and blue. However, further exploration of the effects of different colors, background compatibility, and the effect of gender on this issue is needed. Also, using smaller cues can reduce fatigue. Although cue patterns were effective in reducing fatigue by enhancing attractiveness and diversity, there were no significant distinctions among checkerboard, horizontal grating, and vertical grating patterns. Similarly, no notable differences were detected between square and circular cue comfort scores. Background variations like black screens, white noise, and videos were explored, yet no significant variations emerged between different background scenarios. While earlier research explored diverse stimulation patterns to investigate user fatigue, it is suggested that alongside fatigue reduction, frequency recognition accuracy should be prioritized.

Frequency indicators show inconsistent behaviors among studies, which makes meta-analysis necessary to evaluate these behaviors. The meta-analysis highlighted the increase in α, θ, and $\frac{\alpha +\theta }{\beta }$ frequency band amplitudes as subjects become fatigued. SNR also displayed consistent behavior as a fatigue indicator, but its computation requires knowledge of the stimulation frequency. The application of entropy as a fatigue index, especially in SSVEP-based BCI, was introduced as an alternative that does not depend on stimulation frequency.

In this study, for the first time, a systematic review and meta-analysis were performed on quantitative fatigue indices and the effect of stimulation patterns on fatigue. This study assists researchers in designing a BCI system that causes less user fatigue. According to previous research, the behavior of fatigue indicators differs when the user becomes fatigued; however, the meta-analysis method has revealed which of the indicators has the best performance for detecting fatigue in BCI systems.

## Data availability statement

The original contributions presented in the study are included in the article/supplementary materials, further inquiries can be directed to the corresponding author.

## Author contributions

MA: conceptualization, methodology, formal analysis, software, validation, writing – original draft, and visualization. AM: investigation, conceptualization, software, validation, methodology, resources, writing – review and editing, and supervision. All authors contributed to the article and approved the submitted version.
